# Supplementary material for: Neural Signals Associated with Orienting Response and Arousal Inhibition in Concealed Information Test
Source: Behav Sci (Basel). 2024 Jul 23;14(8):627. doi: 10.3390/bs14080627 (PMC11351989; doi:10.3390/bs14080627)
Supplement: Supplementary file 1 [file behavsci-14-00627-s001.zip › behavsci-3003939-supplementary.pdf]

Supplementary Materials:

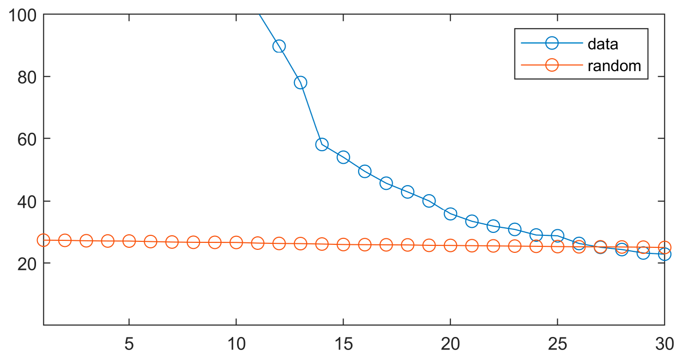

Figure S1: The Scree plot of temporal PCA. We utilized a parallel test to compare the Scree plot of the dataset with that of a completely random dataset. Adjust the scaling (on the left) to 30 to identify the point where the blue line intersects with the blue line. In this instance, 26 factors are indicated as the number (the last point above the line).

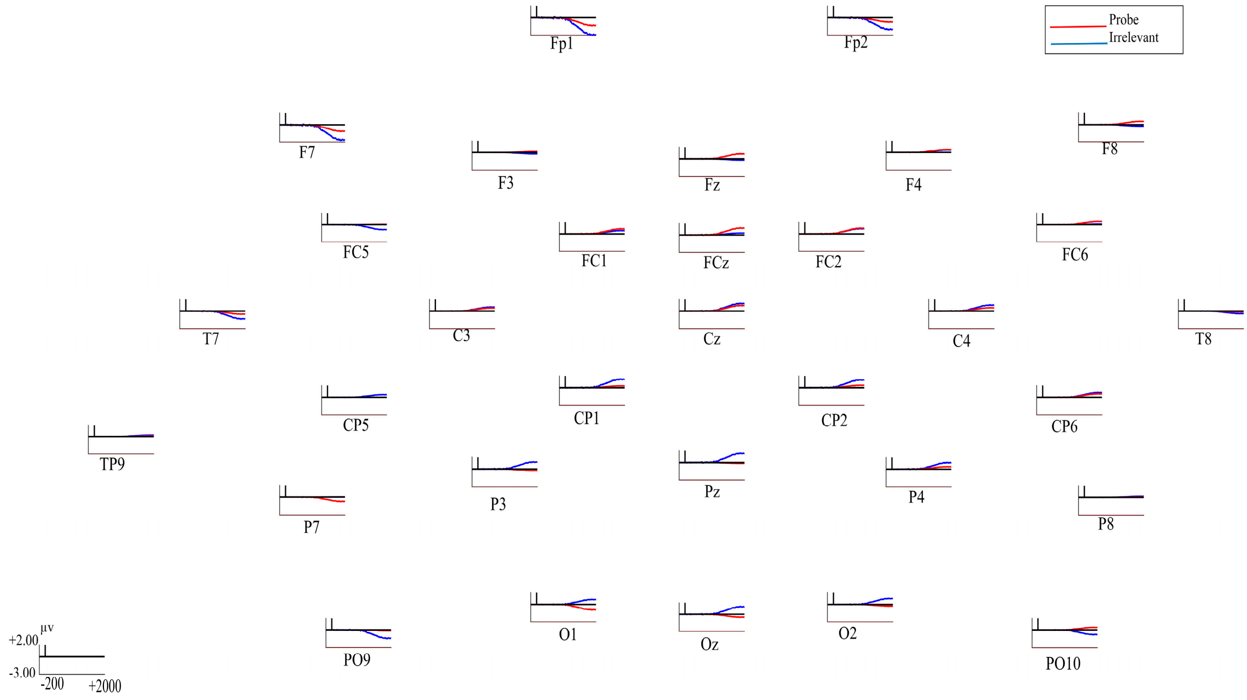

Figure S2: Grand mean (N=40) ERP waveforms of TF01 (–200 to 2000ms, 200 ms pre-stimulus baseline) at 32 electrodes for probe(blue lines) and for irrelevant items (red lines).

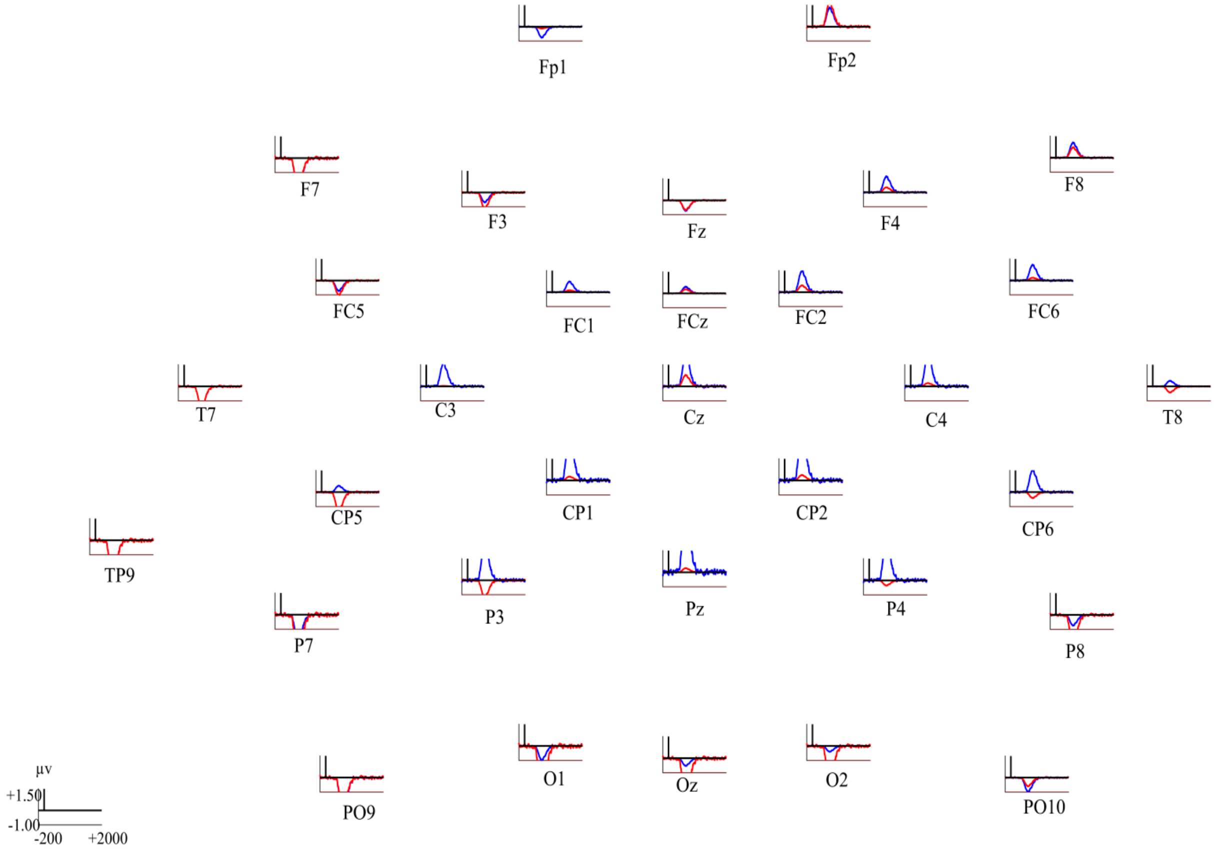

Figure S3: Grand mean (N=40) ERP waveforms of TF02 (−200 to 2000ms, 200 ms pre-stimulus baseline) at 32 electrodes for probe(blue lines) and for irrelevant items (red lines).

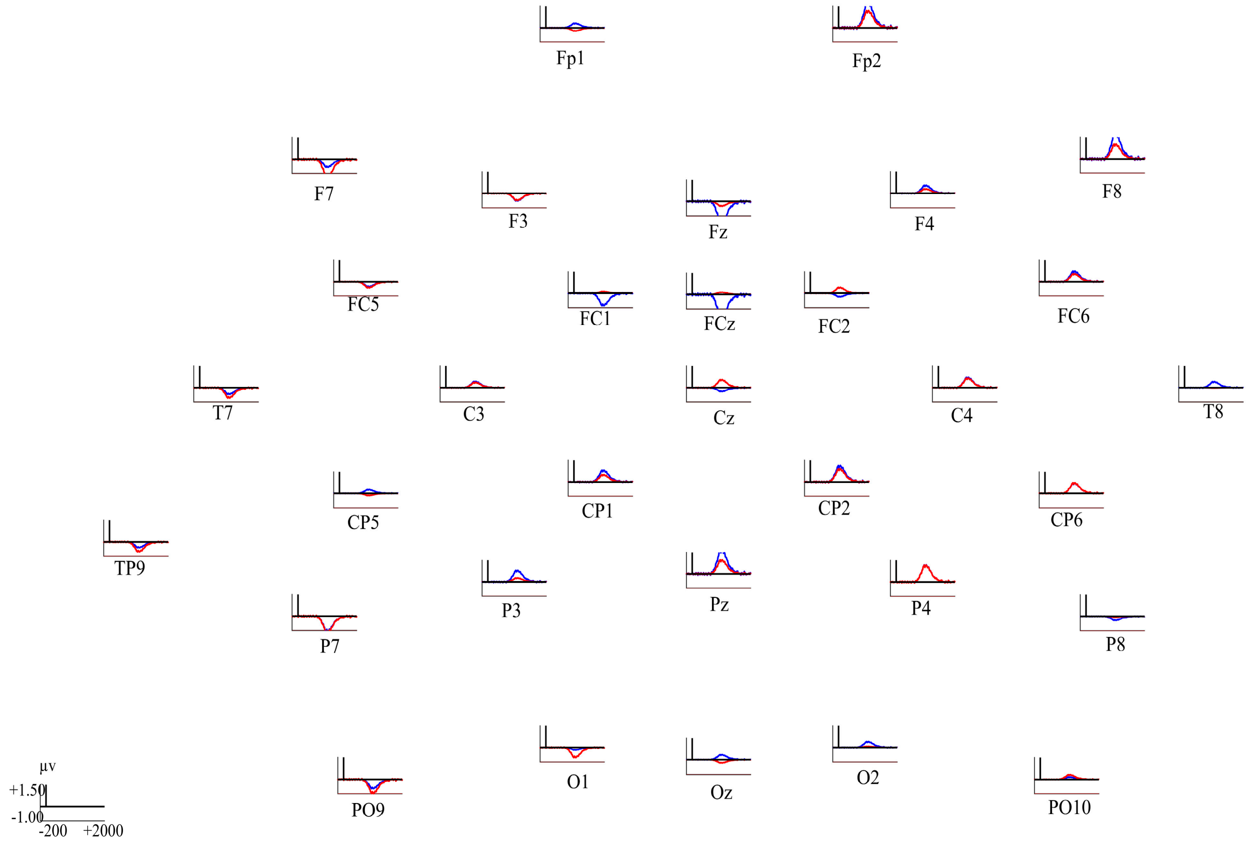

Figure S4: Grand mean (N=40) ERP waveforms of TF03 (−200 to 2000ms, 200 ms pre-stimulus baseline) at 32 electrodes for probe(blue lines) and for irrelevant items (red lines).

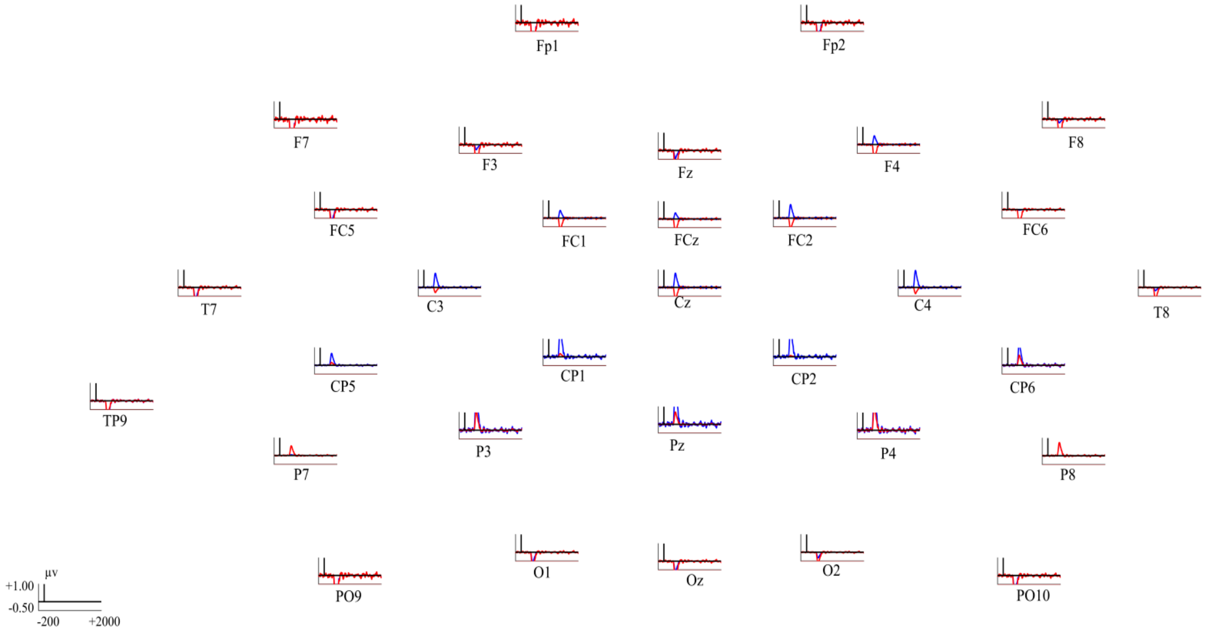

Figure S5: Grand mean (N=40) ERP waveforms of TF04 (−200 to 2000ms, 200 ms pre-stimulus baseline) at 32 electrodes for probe(blue lines) and for irrelevant items (red lines).
